# Elimination of *Onchocerca volvulus* Transmission in the Huehuetenango Focus of Guatemala

**DOI:** 10.1155/2012/638429

**Published:** 2012-08-23

**Authors:** Nancy Cruz-Ortiz, Rodrigo J. Gonzalez, Kim A. Lindblade, Frank O. Richards, Mauricio Sauerbrey, Guillermo Zea-Flores, Alfredo Dominguez, Orlando Oliva, Eduardo Catú, Nidia Rizzo

**Affiliations:** ^1^Centro de Estudios en Salud, Universidad del Valle de Guatemala (UVG), 18 avenida 11-95 Zona 15, Vista Hermosa III, Guatemala City, Guatemala; ^2^Division of Parasitic Diseases and Malaria, Centers for Disease Control and Prevention (CDC), 1600 Clifton Road NE A-06, Atlanta, GA 30333, USA; ^3^CDC Regional Office for Central America and Panama, UVG, 18 avenida 11-95 Zona 15, Vista Hermosa III, Guatemala City, Guatemala; ^4^Carter Center, 453 Freedom Parkway, Atlanta, GA 30307, USA; ^5^Onchocerciasis Elimination Program for the Americas, 14 Calle 3-51 Zona 10, Edificio Murano Center, Oficina 1401, Guatemala City, Guatemala; ^6^Ministerio de Salud Pública y Asistencia Social, 6 avenida 3-45 Zona 11, Guatemala City, Guatemala

## Abstract

In Latin America, onchocerciasis is targeted for elimination by 2012 through twice-yearly mass treatment of the eligible population with ivermectin. In Guatemala, two of the four historical endemic foci have demonstrated elimination of transmission, following World Health Organization guidelines. Using established guidelines ophthalmological, serological, and entomological evaluations were conducted in 2007-8 to determine the transmission status of onchocerciasis in the Huehuetenango focus. The prevalence of *Onchocerca volvulus* microfilariae in the anterior segment of the eye in 365 residents was 0% (95% confidence interval [CI] 0–0.8%), the prevalence of infection of *O. volvulus* in *Simulium ochraceum* among 8252 flies collected between November 2007 and April 2008 was 0% (95% CI 0–0.02%), and the prevalence of antibodies to a recombinant *O. volvulus* antigen in 3118 school age children was 0% (95% CI 0–0.1%). These results showed transmission interruption; thus, in 2009 mass treatment was halted and posttreatment surveillance began. To verify for potential recrudescence an entomological evaluation (from December 2010 to April 2011) was conducted during the 2nd and 3rd year of posttreatment surveillance. A total of 4587 *S. ochraceum* were collected, and the prevalence of infection of *O. volvulus* was 0% (95% CI 0–0.04%). Transmission of onchocerciasis in the Huehuetenango focus has been eliminated.

## 1. Introduction

Onchocerciasis is a vector-borne parasitic disease caused by infection with the filarial nematode *Onchocerca volvulus* and can result in eye or skin lesions. The parasite is transmitted to humans by certain black flies of the genus *Simulium*. Although adult worms can live for years under the skin in fibrous nodules that are often palpable, morbidity is caused by the body's immune reaction to the microfilariae (mf) that leave the nodules and migrate through the skin and sometimes enter the eye [1]. In 2007, The World Health Organization (WHO) estimated 37 million persons were infected with onchocerciasis in 37 endemic countries (30 in Africa, six in the Americas, and one in the Arabian Peninsula) [2]. 

In the Americas there are 13 geographically isolated endemic foci found within Mexico, Guatemala, Colombia, Venezuela, Brazil, and Ecuador [3, 4] where 470,222 persons were at risk of infection in 2011 [5]. The Onchocerciasis Elimination Program of the Americas (OEPA) was established in 1992 in response to a resolution of the Directing Council of the Pan American Health Organization (PAHO) calling for the elimination of onchocerciasis ocular morbidity in the Americas by 2007 [6]. A new resolution in 2008 by PAHO called for the interruption of transmission throughout the region by 2012 [7]. A subsequent 2009 PAHO Resolution (CD49.R19), calling for the elimination or drastic reduction of 12 neglected infectious diseases of poverty in the Americas by 2015, includes onchocerciasis as an elimination target [8]. The OEPA strategy is to support national programs in the six endemic countries to provide twice-yearly mass drug administration (MDA) of ivermectin (Mectizan, donated by Merck & Co.) to ≥85% of the eligible population at risk [6, 9, 10].

**Figure 1:**
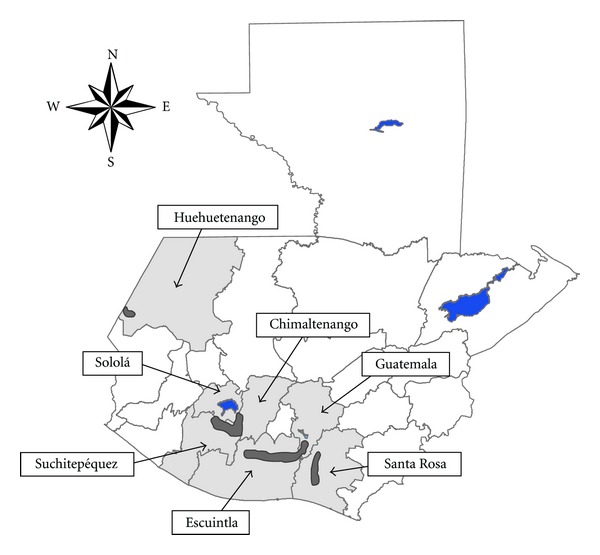
Onchocerciasis foci in Guatemala (in dark gray) in the endemic departments (light gray).

There are four endemic onchocerciasis foci within Guatemala (Figure 1). The Guatemala Ministry of Public Health (MOPH), with the assistance of OEPA, began MDA with ivermectin in 1996 and has achieved ≥85% coverage of the eligible population at risk in twice-yearly MDA rounds since 2002. Interruption of transmission was demonstrated in two of the four foci (Santa Rosa in 2007 and Escuintla-Guatemala in 2008) [11, 12]; in 2010 both foci completed the posttreatment surveillance, and the evaluations showed elimination of transmission [5]. The Huehuetenango focus has participated in 22 rounds of MDA with ivermectin over the past 13 years, with ≥85% coverage in 17 consecutive rounds of twice-yearly ivermectin treatment. E. W. Cupp and M. S. Cupp [9] calculated that the death of adult worms is accelerated in the presence of ivermectin and that the adult population should die (in the absence of ongoing transmission) after 6.5 years (13 rounds) of twice-yearly treatment. Thus, the treatment period appears more than adequate to have eliminated the parasite in the focus of Huehuetenango. We, therefore, report the assessment of the status of 2007-2008 pretreatment and 2010-2011 posttreatment onchocerciasis transmission evaluations in the Huehuetenango focus. 

## 2. Methods

### 2.1. Evaluation Area

**Figure 2:**
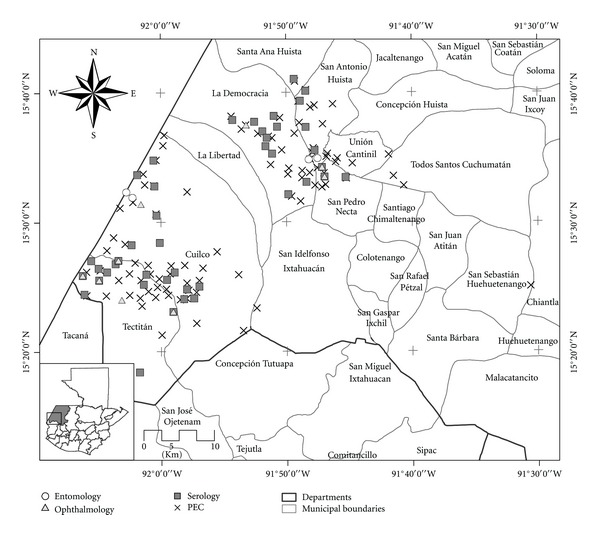
Map of the evaluation area in the Department of Huehuetenango, Guatemala.

The Huehuetenango focus (15.35°N, 91.90°E) is situated in the western highlands of Guatemala along the border with Mexico and consists of eight historically endemic *municipios* (similar to counties) (Figure 2). Historical data from this area showed a gradual reduction of infection prevalence well before the initiation of MDA. Yamagata et al. [13], in an analysis of nodule prevalence data gathered since 1940 by MOPH nodulectomy teams working in the area, showed that nodule prevalence decreased from 41% (in 1940) to 21% in 1969. With the launching of ivermectin MDA, surveys conducted by the MOPH in 1987 and 1992 showed that the prevalence of nodules was 1.1% and 2.2%, respectively (F. O. Richards, Jr., unpublished data). The first surveys to measure the prevalence of infection with *O. volvulus* mf using skin snips in Huehuetenango demonstrated a prevalence of <1% (0.6% in 1987 and 0.8% in 1992). In 2006 an evaluation conducted by the MOPH reported a prevalence of 0% for skin mf and 0.4% for the presence of suspected onchocerciasis nodules (A. Dominguez, pers. comm). 

**Figure 3:**
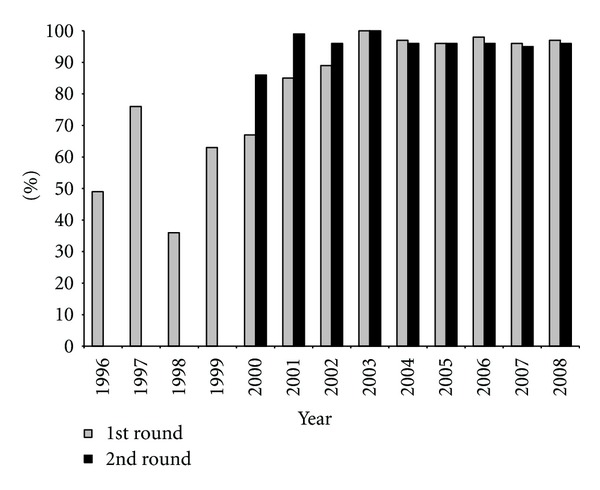
Ivermectin coverage of the eligible population at risk in the Huehuetenango focus from 1996 to 2008.

Annual MDA with ivermectin was launched in the endemic *municipios* of Huehuetenango in 1996 (Figure 3). The program was strengthened in 2000, when twice per year treatment was instituted, and ≥85% coverage of the eligible population at risk was achieved for the first time. In 2008, there were 30,239 persons at risk of transmission, and the eligible population (nonpregnant, healthy, and older than five years of age) to receive treatment with ivermectin numbered 27,797. Between 2000 and 2008, there was sustained and adequate coverage (≥85% treatment coverage) for 17 consecutive twice-yearly MDA rounds. 

### 2.2. Identification of Potentially Endemic Communities

Using historical data from the MOPH from 1980 to 1990, we created a list of potentially endemic communities (PEC) for onchocerciasis using at least one of the following characteristics: (a) past evidence of onchocerciasis transmission (presence of at least one resident of the community with a palpable nodule and/or mf in the skin), (b) suspicion of past transmission (communities where a nodule survey took place, even if no transmission was detected), and/or (c) communities currently participating in MDA with the MOPH. We identified 94 PEC from the eight historically endemic *municipios*. Of these PECs, 43 were participating in MDA at the time of the evaluation; by the mid-1990s, the MOPH had removed the remaining communities from the list of endemic communities as they showed no evidence of infection. 

### 2.3. Evaluation of Transmission Interruption

The evaluation of transmission interruption conducted in Huehuetenango consisted of three assessments: ophthalmological, serological and entomological. These assessments were conducted following the 2001 WHO guidelines as modified by Lindblade et al. [11], with the following criteria indicating that transmission had been interrupted: (1) <1% prevalence of mf in the anterior segment of the eye, (2) <0.1% cumulative incidence of infection in children (measured serologically), and (3) absence or near absence of infective (L3) blackflies (<1 infective fly per 2000 tested).

#### 2.3.1. Ophthalmological Evaluation

To determine the presence of ocular morbidity associated with onchocerciasis, we measured the prevalence of mf in the anterior segment (MfAS) of the eye in a sample of residents from the PEC in Huehuetenango. A minimum sample size of 300 persons was needed to calculate a one-sided 95% confidence interval (CI) around a prevalence of zero that would exclude 1% [11]. Assuming a 15% nonresponse rate, our final sample size was 353. To increase the likelihood of finding cases with ocular morbidity, we identified all PECs (*n* = 19) where the prevalence of mf in the skin was >0% in any survey conducted between 1987 and 1992. Due to access problems, distance between communities, availability of the project ophthalmologist, and the number of persons who could be examined in one day, it was considered feasible to sample 40 residents from each of nine communities, which were selected from among the 19 eligible communities by probability proportional to size sampling. All nine selected communities were mapped and a census conducted on all households using a personal digital assistant (PDA) to identify all eligible persons (residents ≥7 years of age with at least five years living in the community) for the evaluation. We selected 13 houses (estimating three eligible individuals per household) at random from each of the selected communities after mapping, and all eligible individuals from these households were invited to participate in the evaluation. 

An ophthalmologist with extensive experience in onchocerciasis-related ocular morbidity examined participants using a method described previously [11, 14]. Briefly, residents were examined with a slit lamp for the presence of microfilaria in the anterior chamber or the cornea of either eye. 

#### 2.3.2. Serological Evaluation

The cumulative incidence of infection with *O. volvulus* was determined serologically by measuring the prevalence of IgG4 antibodies to the recombinant antigen OV16 in school age children (6 to 12 years old) using methods previously described [11, 15]. Briefly, a sample size of 3000 children was required; assuming a 30% non-response rate, our final sample size was 4286 children. All schools in the PEC were identified, and the number of enrolled children 6 to 12 years old determined. The schools were ordered at random and then selected in order until the required sample size was achieved. Forty-three schools from 37 PECs (seven *municipios*) were selected by this procedure. All children aged 6 to 12 from each selected school were asked to participate in the evaluation, and children that were not present at school during the evaluation were followed up at their homes and asked to participate. 

Finger-prick, filter paper blood samples were collected from all participating children as previously described [11]. IgG4 antibodies to the recombinant antigen OV16 were determined using the enzyme-linked immunosorbent assay (ELISA) method described previously [11, 15]. 

#### 2.3.3. Entomological Evaluation

Black flies were collected twice per month from November 2007 to April 2008 (the peak black fly biting season), in four coffee plantations located in the same geographical areas of the PECs. Plantations were purposively selected to find those with high densities of *S. ochraceum* and owners willing to participate in the evaluation. We used a standard black fly collection method described in previous studies [11, 16]. Briefly, in each plantation, four collection sites, two near the residences and two in the coffee plantation, were identified. A team, comprised of a collector and a paid human attractant (male resident from 18 to 50 years of age), was employed at each collection site and worked two days per month per site. All human attractants received a dose of ivermectin before the evaluation and were assessed for the presence of antibodies to *O. volvulus* using the OV16 ELISA test before and after the evaluation. Flies were pooled and evaluated by a standard polymerase chain reaction (PCR) method to identify *O. volvulus* DNA [17–19]. 

### 2.4. Evaluation of Transmission Elimination

 The evaluation of transmission elimination was conducted between the second and third year after treatment suspension, during peak black fly biting season. This evaluation consisted only of the entomological assessment since *O. volvulus* recrudescence should first be detected in the vectors [20]. The criteria to determine elimination of transmission is the absence or near absence of infective blackflies (<1 infective fly per 2000 tested).

#### 2.4.1. Entomological Evaluation

Collection of backflies was carried out from December 2010 to April 2011 in the same four coffee plantations as the pretreatment evaluation and following the same procedures. 

### 2.5. Statistical Analysis

The exact one-sided 95% CI for the prevalence of MfAS and IgG4 antibodies to OV16 was obtained as previously described [11] using SAS software (version 9.0, SAS Institute, Cary, NC, USA). The proportion of infective flies and the 95% CI were calculated using Poolscreen 2.0 [17, 18, 21]. Standard procedures were used to obtain geometric mean of the biting rate, arithmetic mean of the biting rate, biting density, and the seasonal transmission potential [11]. The seasonal transmission potential (STP) was calculated as the product of the seasonal biting density (SBD; number of bites per person during the transmission season), the proportion of flies with infective-stage *O. volvulus* larvae (PCR positive), and the mean number of infective larvae per infective fly (assumed to be one in an area of low transmission). Entomological analyses were performed using R 2.13.1 (2011-07-08) [22].

### 2.6. Human Subjects

The protocol for these evaluations was reviewed and approved by the Centers for Disease Control and Prevention (Atlanta, GA, USA), the Universidad del Valle de Guatemala (Guatemala City, Guatemala), and the Guatemala MOPH. Written informed consent was obtained from participants 18 years of age and older; parents or guardians of children less than 18 years provided written, informed consent for their participation. All children were asked to sign an assent form for participation. 

## 3. Results

### 3.1. Evaluation of Transmission Interruption

Ophthalmological, serological, and entomological evaluations were conducted from June 2007 to April 2008. The results from these evaluations showed the transmission status of *O. volvulus* in the Huehuetenango focus, after 14 rounds of ivermectin treatment with coverages higher than 85% (Figure 3).

#### 3.1.1. Ophthalmological Evaluation

The ophthalmological evaluation was carried out from 17 to 28 September 2007. A total of 365 eligible residents from nine selected PECs were evaluated.  Table 1 shows the nine PECs with the eligible population and the number of individuals evaluated in each PEC (Figure 2). Of these individuals, 82% reported having lived in the community throughout their life, 55% were female, and 64% were between the age of seven and 29. Ophthalmological evaluation showed that 97% of the participants had a visual acuity between 20/20 and 20/70. No MfAS were found in any of the participants. The prevalence of MfAS was 0% (95% CI 0–0.8%). 

#### 3.1.2. Serological Evaluation

Serological evaluation was carried out from June to July 2007. We identified 3910 children aged from 6 to 12 years old enrolled in the 43 selected schools (Table 2), of which 3118 (80%) participated in the evaluation. The mean age was 9.1 years (SD 2.1 years), 52% were male, and the average of years of living in the community was 9.3 years (SD 1.8 years). None of the children tested were found to be seropositive, resulting in a cumulative incidence of 0% (95% CI 0–0.1%).

#### 3.1.3. Entomological Evaluation

Entomological evaluation was carried out in four coffee plantations (two located in Agua Dulce, Cuilco and two in Marilandia, San Pedro Necta) which presented high densities of *S. ochraceum*. Thirty nine collections days, equivalent 1220 hours of collection, were completed between November 2007 and April 2008 (Table 3). A total of 8252 *S. ochraceum* and 11473 S. metallicum were collected As reported before [23], the biting density of *S. ochraceum* is higher in December and starts to decrease in February (Table 3). Overall, the geometric mean biting rate was 3.2 (95% CI 2.9–3.5) bites/person/hour, with the highest biting rate in November (16.7 bites/person/hour) and the lowest in April (0.7 bites/person/hour). The SBD indicating the total number of bites per person per season was 5765 (95% CI 5263–6300). The paid human attractants were tested at the beginning and end of the evaluation for antibodies to OV-16, but none was found positive.

The *S. ochraceum* collected were grouped in 357 pools, and all were negative for *O. volvulus* infection. The prevalence of infection was 0% (95% CI 0–0.02%), and the maximum prevalence of infection was estimated to be 0.4/2000 flies. The STP was 0 infective larvae/person/season, but the maximum potential STP (using the upper boundary on the prevalence of infection in *S. ochraceum*) was 1.3 infective larvae/person/season. 

### 3.2. Evaluation of Transmission Elimination

The results from the previous evaluations showed that transmission of *O. volvulus* through *S. ochraceum* was interrupted, thus in 2009 ivermectin MDA was suspended from the Huehuetenango focus. Following WHO guidelines, from December 2010 to April 2011, we conducted the entomological evaluation to evaluate the transmission of *O. volvulus* after treatment suspension.

#### 3.2.1. Entomological Evaluation

The entomological evaluation was conducted in the same four coffee plantations as the previous entomological evaluation. Forty collection days corresponding to 1280 hours of collection were conducted. A total of 4587 *S. ochraceum* and 4912 *S. metallicum* were collected (Table 4), and a significant reduction in the density of both species was observed when compared to the 2007-2008 collection. However the biting density pattern of *S. ochraceum* was similar as that observed in 2007-2008. The overall geometric mean biting rate was 1.8 (95% CI 1.6–2.0) bites/person/hour, and the SBD was 2693 bites/person (95% CI 2454–2945). The paid human attractants did not present antibodies to OV16 before or after the evaluation.

The *S. ochraceum* collected were grouped in 147 pools, and all were negative for *O. volvulus* infection. The prevalence of infection was 0% (95% CI 0–0.04%), and the maximum prevalence of infection was estimated to be 0.8/2000 flies. The STP was 0 infective larvae/person/season, with the maximum potential STP (using the upper boundary on the prevalence of infection in *S. ochraceum*) being 1.1 infective larvae/person/season. As a result of low number of *S. ochraceum* collected, all *S. metallicum* were grouped in pools in the same manner as described for *S. ochraceum* and processed by PCR to determine if they were infected with *O. volvulus*. All *S. metallicum* were negative for *O. volvulus*; thus, there are no parasites circulating in these black flies. 

## 4. Discussion

In 2001, the WHO established a set of guidelines to assist onchocerciasis programs to determine whether interruption of transmission had occurred and MDA with ivermectin could be stopped [16]. The process outlined by WHO involves four phases: (1) suppression of transmission, where new infective stage larvae are no longer introduced into the human population by the vectors, but the parasite population maintains the ability to recover if interventions are withdrawn; (2) interruption of transmission, when the parasite population is thought to be unable to recover and interventions (in this case, twice-yearly ivermectin treatment) can be halted; (3) precertification, during which time posttreatment surveillance is needed for three years with an in depth evaluation to take place during the second or third year, depending on the peak transmission season [20]; (4) if this evaluation was negative, a declaration of elimination. When all foci within a country reach the final phase, the country may request certification of elimination of onchocerciasis from WHO [16]. 

We conducted an assessment of the status of *O. volvulus* transmission in the third onchocerciasis focus of Guatemala to be so evaluated by this process. Data from evaluations conducted by the MOPH in Huehuetenango in the 1990s indicated that the parasite had, at best, a tenuous hold when MDA was launched in 1996. Our results in 2007-2008 confirmed that transmission of onchocerciasis had been successfully interrupted in Huehuetenango after 22 rounds of MDA over 13 years. We found no evidence of ocular lesions attributed to *O. volvulus* infection. Similarly, we found no serological evidence of recent exposure to the parasite among 6–12-year-old children residing in the endemic area, nor any entomological evidence of infected or infective black fly vectors. The maximum STP in this area was conservatively calculated at 1.2 infective larvae/person/season, which is not sufficient to sustain transmission in *S. ochraceum* areas [24]. The expert steering committee of OEPA (the Program Coordinating Committee (PCC)) reviewed the results of this evaluation and formally recommended to the Guatemala MOPH that MDA with ivermectin be suspended in Huehuetenango, beginning in 2009. This recommendation was accepted, and Huehuetenango joined the Santa Rosa and Escuintla-Guatemala foci in the posttreatment surveillance period. 

One challenge to maintaining the Huehuetenango focus free of onchocerciasis is its proximity to the border with Mexico (Figure 1) and, in particular, to the South Chiapas onchocerciasis focus [19]. There has been conjecture that the two foci were linked through migrant coffee workers moving between the two countries and acquiring onchocerciasis in one or both foci. The unlikelihood of migrant workers being able to maintain onchocerciasis transmission in Guatemala and Mexico has been studied and discussed elsewhere [25, 26]. Of the two foci, South Chiapas historically had the highest levels of active onchocerciasis transmission in all of Mexico [19] whereas the evidence from Huehuetenango during the same time period showed significant decreases in onchocerciasis prevalence even before the MDA program, suggesting that South Chiapas had little effect on transmission in Huehuetenango. An MDA program requiring treatment four times per year was required to interrupt transmission in the South Chiapas focus; at the beginning of 2012 MDA was suspended and post treatment surveillance initiated there [27], thus it is unlikely that onchocerciasis could be reintroduced into Huehuetenango from Mexico. 

Following PTS guidelines [20], from December 2010 to April 2011 an entomological evaluation was conducted in Huehuetenango to verify that *O. volvulus* transmission had not recrudesced. The results from this evaluation showed that *O. volvulus* L3 are not circulating in the primary vector *S. ochraceum* or in potentially a secondary vector, *S. metallicum*. A conservative calculation of STP using upper 95% confidence interval values for *S. ochraceum* was 1.1 infective larva/person/season, which is insufficient to maintain the parasite population. Thus we conclude that *O. volvulus* transmission has been eliminated from the Huehuetenango focus. Currently in Guatemala the three hypoendemic foci (Santa Rosa, Escuintla-Guatemala and Huehuetenango) have demonstrated, through completion of PTS, elimination of transmission. The only focus that is currently under post treatment surveillance is the hyperendemic central endemic focus, which only stopped MDA at the beginning of 2012. When the Central Endemic focus successfully completes PTS and attains elimination status, then the entire country of Guatemala will be able to request WHO certification of elimination in 2015. 

In May 2009, the US President Barack Obama announced a new Global Health Initiative (GHI) to improve health outcomes in partner countries. Neglected tropical diseases feature prominently in the GHI, and a key target is elimination of onchocerciasis from the Americas (http://www.america.gov/st/texttrans-english/2010/August/20100817134101su0.3731152.html, accessed on September 1, 2010). As the elimination of onchocerciasis from the Americas by 2015 looks to be on target, this could be one of the early successes of the GHI and a significant public health success story that can help motivate and inform other elimination efforts. 

## Figures and Tables

**Table 1:** Potentially endemic communities included in the ophthalmological evaluation. Nine communities were selected to carry out the ophthalmological evaluation, and a total of 10,183 persons were eligible to participate, of whom 365 were evaluated.

Community	*Municipio*	Eligible population	Population evaluated
Michicoy	San Pedro Necta	839	49
Ixnul	San Pedro Necta	1201	45
Santa Rosa	Cuilco	1550	41
Agua Dulce	Cuilco	2331	50
El Rodeo	Cuilco	1113	43
Chiquihuil	Cuilco	726	36
Union Frontera	Cuilco	194	42
Cabecera Municipal Democracia	La Democracia	1913	24
El Tablon	Cuilco	316	35

Total		10,183	365

**Table 2:** Potentially endemic communities included in the serological evaluation. Forty-three schools were survey for the serological evaluation. A total of 3910 children were eligible to participate.

School	Community	*Municipio*	Eligible children	Children sampled
1	Cumil	Cuilco	145	139
2	El Astillero	Cuilco	105	84
3	El Rodeo	Cuilco	111	53
4	El Zapotillo	Cuilco	44	34
5	La Laguna	Cuilco	76	71
6	Sabunul	Cuilco	179	121
7	Sosi	Cuilco	149	118
8	Yulva	Cuilco	218	193
9	Plan de las Vigas	Cuilco	46	41
10	Ampliacion Nueva Reforma	Cuilco	60	44
11	El Boqueron	Cuilco	103	63
12	Buenos Aires	Cuilco	32	25
13	Carrizal	Cuilco	63	63
14	Chiquihuil	Cuilco	133	100
15	Cruz de Pinapa	Cuilco	109	69
16	Ixtatilar	Cuilco	43	33
17	Jalapa	Cuilco	64	53
18	Cruz Miramar	Cuilco	33	29
19	La Soledad	Cuilco	58	58
20	Los Garcia	Cuilco	41	35
21	Salitre	Cuilco	17	17
22	Tablon (Sosi)	Cuilco	64	57
23	Union Frontera	Cuilco	36	36
24	Union Batal	Cuilco	118	109
25	Buena Vista	La Democracia	236	228
26	Cementerio	La Democracia	111	88
27	Norte	La Democracia	49	40
28	El Chorro	La Democracia	24	14
29	La Montañita	La Democracia	76	70
30	La Vega	La Democracia	177	130
31	Nuevo San Rafael	La Democracia	85	33
32	Plan Grande	La Democracia	67	54
33	Cerro Verde	La Libertad	123	85
34	Nojoya	San Antonio Huista	69	60
35	El Pajal	San Antonio Huista	84	40
36	Tablon Viejo	San Antonio Huista	31	29
37	Isnul	San Pedro Necta	183	181
38	Michicoy	San Pedro Necta	97	74
39	Rio Ocho	San Pedro Necta	155	71
40	Jolimex	San Pedro Necta	49	35
41	El Pajarito	San Pedro Necta	53	53
42	Finca Santa Cecilia	San Pedro Necta	35	35
43	Cabecera Departamental	Santa Ana Huista	159	153

Total			3910	3118

**Table 3:** Entomological collections in the Huehuetenango focus (from November 2007 to April 2008).

Month	Hours collected	Days collected	Simulium ochraceum			*Simulium metallicum*
			Number captured	Biting rate per hour^∗^	Biting density^§^	Number captured
November	57	2	1641	16.7 (11.4–24.2)	5019 (3432–7270)	796
December	160	5	2745	12.7 (10.8–15)	3951 (3346–4655)	2639
January	252	8	1558	4.5 (3.9–5.1)	1381 (1194–1589)	3020
February	256	8	1083	2.5 (2.1–3)	726 (602–865)	2025
March	252	8	963	1.9 (1.6–2.4)	600 (483–732)	1753
April	243	8	262	0.7 (0.5–0.8)	203 (158–252)	1240

Total	1220	39	8252	3.2 (2.9–3.5)	5765 (5263–6300)	11473

*Geometric mean of biting rate per hour (95% CI).

^§^Number of black fly bites per person per month (geometric mean biting rate per hour ×10 hours per day × number of days in a month) (95% CI).

**Table 4:** Entomological collections in the Huehuetenango focus (from December 2010 to April 2011).

Month	Hours collected	Days collected	*Simulium ochraceum*			*Simulium metallicum*
			Number captured	Biting rate per hour^∗^	Biting density^§^	Number captured
December	224	7	1743	4.2 (3.5–5.1)	1309 (1075–1581)	1603
January	288	9	1230	2.5 (2.1–2.9)	769 (650–903)	911
February	256	8	603	1.5 (1.2–1.8)	420 (347–500)	1055
March	256	8	775	1.6 (1.3–2)	501 (403–611)	864
April	256	8	236	0.4 (0.3–0.6)	130 (93–170)	479

Total	1280	40	4587	1.8 (1.6–2)	2693 (2454–2945)	4912

*Geometric mean of biting rate per hour (95% CI).

^§^Number of black fly bites per person per month (geometric mean biting rate per hour ×10 hours per day × number of days in a month) (95% CI).
